# Activation of Nrf2 Attenuates Pulmonary Vascular Remodeling via Inhibiting Endothelial-to-Mesenchymal Transition: an Insight from a Plant Polyphenol: Erratum

**DOI:** 10.7150/ijbs.96249

**Published:** 2024-03-21

**Authors:** Yucai Chen, Tianyi Yuan, Huifang Zhang, Yu Yan, Danshu Wang, Lianhua Fang, Yang Lu, Guanhua Du

**Affiliations:** 1 State Key Laboratory of Bioactive Substances and Functions of Natural Medicines;; 2 Beijing Key Laboratory of Drug Targets Identification and Drug Screening;; 3 Beijing Key Laboratory of Polymorphic Drugs, Institute of Materia Medica Chinese Academy of Medical Sciences and Peking Union Medical College, Beijing 100050, China.

In our paper, the author noticed an error in Figure 2. There is a non-subjective error in the article after examining the original data. As shown in Figure 2D in the article, due to negligence in typesetting, we accidentally placed the representative images originally belonging to the model group into the SAA 0.3 μM group.

We checked the original data again and made sure that the conclusion of the article was not affected by the error. All authors agree to the the erratum. we apologize for any inconvenience caused by the negligence in our work.

Figure 2 should be corrected as follows.

## Figures and Tables

**Figure 2:**
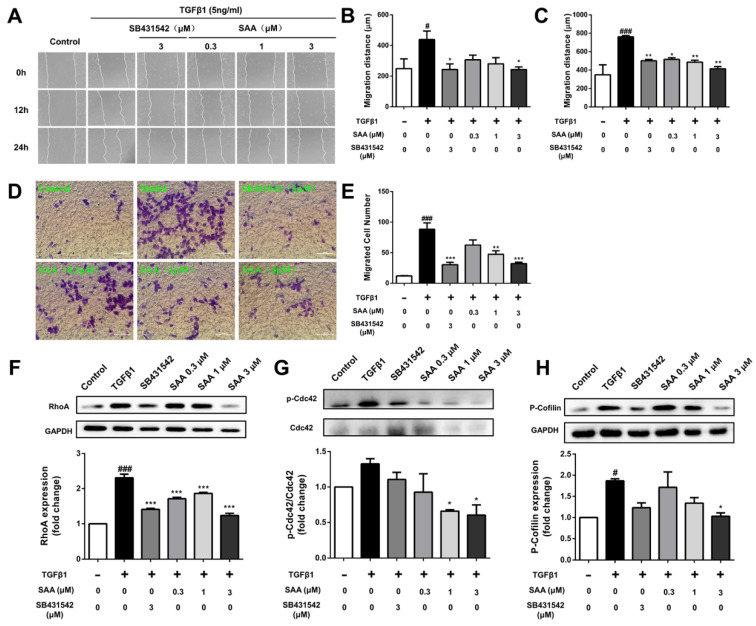
Correct image.

